# Bilateral acute anterior uveitis and optic nerve edema as a manifestation of coronavirus disease‐2019 (COVID‐19): A case report

**DOI:** 10.1002/ccr3.7473

**Published:** 2023-06-07

**Authors:** Aria Mohamadzadeh, Dena Mohamadzadeh

**Affiliations:** ^1^ Faculty of Veterinary Medicine Razi University Kermanshah Iran; ^2^ Clinical Research Development Center, Imam Reza Hospital Kermanshah University of Medical Sciences Kermanshah Iran

**Keywords:** acute anterior uveitis, COVID‐19, MIS, optic disk edema, SARS‐COV‐2

## Abstract

**Key clinical message:**

Acute anterior uveitis and optic disk edema could be a manifestation of COVID‐19 infection, and healthcare providers should be aware of this possible consequence for in‐time diagnosis and treatment.

**Abstract:**

Since the beginning of the coronavirus disease‐2019 (COVID‐19) pandemic, a wide range of clinical manifestations have been associated with this novel infection. The objective of this study was to show that acute anterior uveitis and optic disk edema could be possible manifestations of COVID‐19 infection. The patient was a nine‐year‐old girl presenting with prolonged fever, myalgia, cough, diarrhea, and skin rashes. She also reported blurred vision, Photophobia, and eye redness. PCR test for COVID‐19 returned positive. Imaging investigations showed pleural and pericardial effusion, mediastinal lymphadenopathy, and heart valve regurgitation. She was diagnosed with MIS‐C and treated with methylprednisolone and IVIG. Bilateral acute anterior uveitis and optic disk edema was detected by slit lamp and fundus examination. She was successfully treated and follow‐up ophthalmologic examinations showed improvement.

## Graphical abstract

Chest CT scan of the patient showing bilateral pleural effusion, and subsegmental atelectasis in the context of MIS‐C in a nine‐year‐old girl infected with COVID‐19.

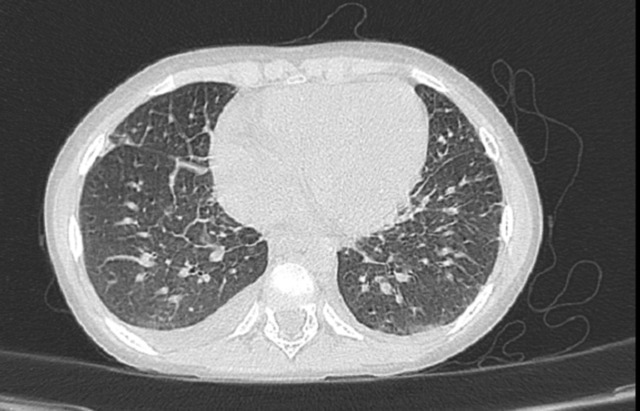

## 1 INTRODUCTION

Coronavirus disease‐2019 (COVID‐19) is a novel infection that first appeared in Wuhan, China. It has been well‐established that different organs including the heart, kidney, pulmonary, and gastrointestinal systems might be involved during or post‐COVID‐19 infection. A possible reason is that the virus enters host cells through the angiotensin‐converting enzyme 2 (ACE 2) receptor, and this receptor is expressed by different human organs.^1^ The angiotensin‐converting enzyme 2 receptor is also expressed in different parts of the eyes including the conjunctiva, cornea, limbus, aqueous humor, and retina.^2^

In addition, COVID‐19‐associated multisystem inflammatory syndrome in children (MIS‐C) can affect different organ systems including skin, renal, cardiac, hematological, and gastrointestinal systems, but ocular involvement is not still a part of the World Health Organization (WHO) criteria or the Center for Disease Control and Prevention criteria for MIS‐C.^3^

Whether uveitis or optic disk edema would be manifestations of acute COVID‐19 infection or they can occur as a part of COVID‐19‐associated MIS‐C is still unknown.

## 2 CASE PRESENTATION

A nine‐year‐old girl presented to the emergency department of Dr. Mohammad Kermanshahi Pediatric Hospital in Kermanshah City, Iran, on September 25, 2021. She complained of fever, myalgia, loss of appetite, headache, cough, diarrhea, abdominal pain, and skin rashes. Her symptoms initiated five or 6 days prior to this hospitalization, and her mother reported that fever was not controlled by acetaminophen syrup in the first 2 days of the onset of symptoms. She also complained of blurred vision, photophobia, pain, and redness of the eyes on the day of hospitalization. Her parents claimed that she had had mild gastrointestinal symptoms such as diarrhea about 4 weeks prior to admission, which lasted for 2 days and resolved completely without specific treatment. She denied eye trauma. Her past medical history and familial history were unremarkable.

On general examination, her vital signs were as follows: pulse rate of 98 beats/min, respiratory rate of 20, blood pressure of 100/65, and she was not febrile on the day of admission. Jaundice, paleness, and cyanosis of the skin were absent. Lymphadenopathy was not detected. Diffuse erythematous patches and plaques were observed on her face, trunk, and limbs. A ciliary injection was detected in her eyes. Eye movements were normal in all directions and were not painful. Pupil's size and their reaction to light were normal. Examination of the respiratory and cardiovascular systems was within normal limits. Abdominal examination was unremarkable.

Laboratory investigation showed hemoglobin of 13.4 mg/dL, white blood cell count (WBC) 8.9 ×10^3^/mm^3^ (differential count: neutrophils 88%, lymphocytes 7%), platelet counts 191 ×10^3^/mm^3^, creatinine 0.9 mg/dL, aspartate transaminase (AST) 16 IU/L (normal: 0–31 IU/L), alanine transaminase (ALT) 20 IU/L (normal: 0–34 IU/L), alkaline phosphatase (ALP) 196 U/L (normal: 64–306 U/L), erythrocyte sedimentation rate (ESR) 26 mm/h, and C‐reactive protein (CRP) were positive. Urine analysis and stool examination were normal. Blood and urine cultures were sterile. PCR (polymerase chain reaction) testing for COVID‐19 was positive.

**FIGURE 1:**
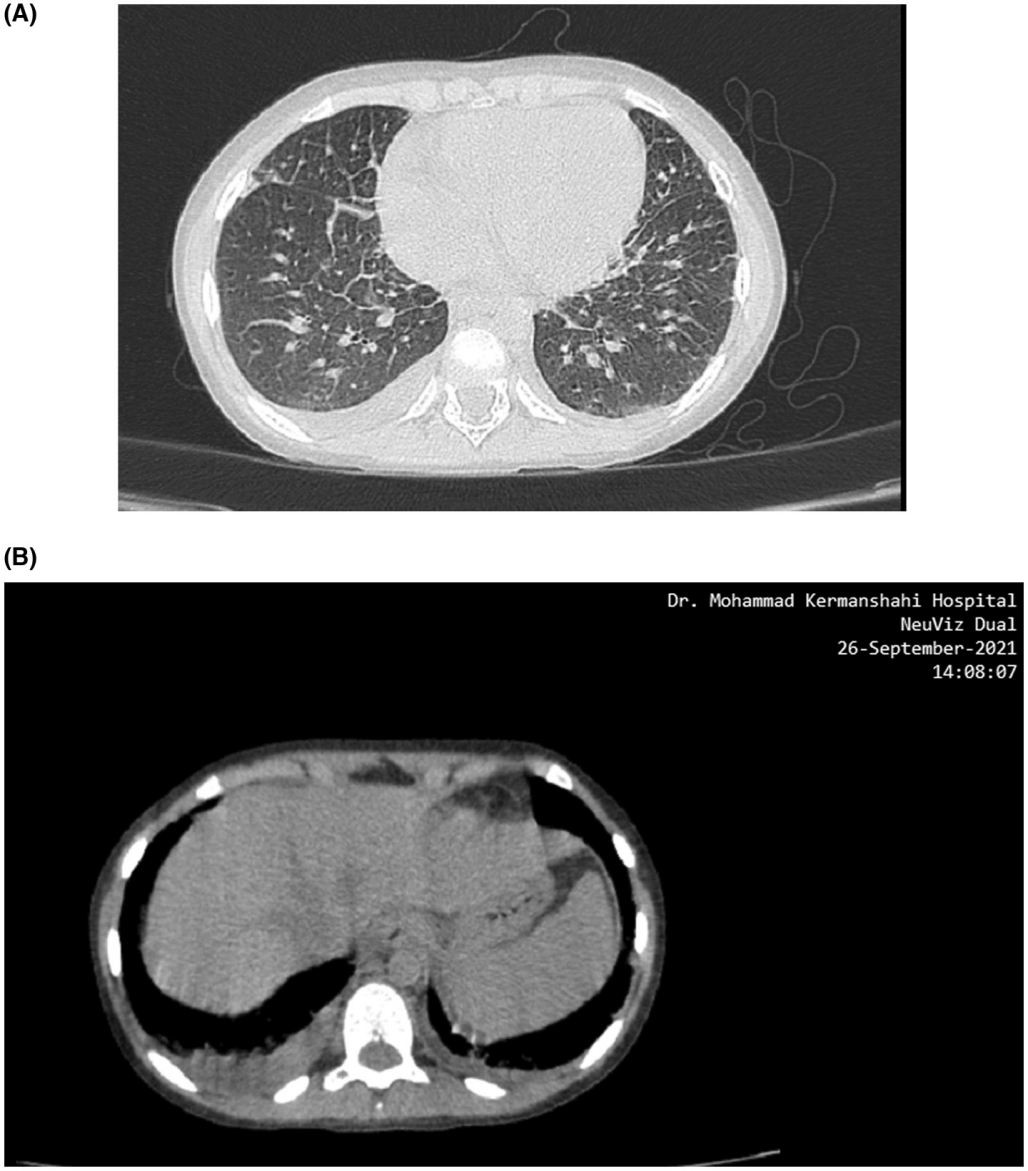
(A, B) Chest CT (computerized tomography) scan showing (A). bilateral pleural effusion (8 mm on right and 4 mm on the left), (B) mediastinal lymphadenopathy (the biggest = 7 mm), and (C) subsegmental atelectasis in the right middle lobe (RML) and right lower lobe (RLL).

Ultrasonography of the abdomen was performed and revealed no pathological finding. Chest CT (computerized tomography) scan showed mild bilateral pleural effusion, mediastinal lymphadenopathy, and subsegmental atelectasis (Figure 1). Echocardiography was remarkable for the pericardial effusion of 6‐millimeter thickness, mild MR (mitral regurgitation), and mild TR (tricuspid regurgitation). MIS‐C (a multisystem inflammatory syndrome in children) was diagnosed based on the MIS‐C diagnostic criteria of both WHO and, the Center for Disease Control and Prevention (the United States)^3^ for the presence of fever, skin rashes, diarrhea, cardiac involvement, respiratory involvement, elevated ESR and CRP, and positive PCR of COVID‐19. She was started on methylprednisolone 30 mg/daily for 5 days followed by oral prednisolone 5 mg/daily and IVIG 2 g/kg in four divided doses. Immunological workup including antinuclear antibodies (ANA), and rheumatoid factor (RF) were within normal limits.

An ophthalmologist visited the patient. Visual acuity was 6/9. Slit lamp examination and indirect ophthalmoscopy were performed. Bilateral acute anterior uveitis was documented. Findings of the slit lamp examination included mild ciliary injection, anterior chamber reaction with 2+ cells, and flares. The posterior chamber was clear. Examination of the fundus showed bilateral optic disk edema. Intraocular pressure (IOP) was unaffected. There was no finding in favor of bacterial or viral infections such as herpetic keratitis. Orbital sonography showed increased thickness of both optic nerves. Corticosteroids (betamethasone eye drop of 0.1% twice daily for 7 days) and cycloplegic eye drops were added to the ongoing treatment. The ocular symptoms improved gradually. In the follow‐up echocardiography 2 weeks later, pericardial effusion decreased to 3 mm. Visual acuity was 6/6. She was discharged in stable condition. Repeated ophthalmologic examinations after discharge showed complete improvement after 4 weeks.

## 3 DISCUSSION

Acute anterior uveitis is the most common type of uveitis and is defined as inflammation of the iris (middle layer of the eyes). While idiopathic uveitis is the major type, various etiologies have been described for uveitis including autoimmune inflammatory systemic disorders (AIDS), infections, side effects of medications, and eye injury.^4^ AIDS‐causing anterior uveitis including HLA B‐27‐associated spondyloarthritis, Juvenile idiopathic arthritis‐associated uveitis, sarcoidosis, systemic lupus erythematosus, Behcet's syndrome, and Crohn's disease.^5^ Viruses have been found to be the commonest infectious agents causing anterior uveitis. Common etiologies of viral anterior uveitis are herpes simplex virus, varicella‐zoster virus, cytomegalovirus, and rubella. Human immunodeficiency virus and human T‐cell lymphotropic virus type 1 are less common etiologies.^6^

Here, we reported a case of bilateral anterior uveitis and optic disk edema with a positive PCR test for COVID‐19 infection. She met the MIS‐C diagnostic criteria of both WHO and, the Center for Disease Control and Prevention (the United States) for the presence of fever, skin rashes, diarrhea, cardiac involvement, respiratory involvement, elevated ESR and CRP, and positive PCR of COVID‐19. As we know MIS‐C leads to multi‐organ involvement, but ocular involvement is not a part of the diagnostic criteria yet.^3^ Bettach et al.^7^ described a 54‐year‐old woman diagnosed with bilateral anterior uveitis. She was hospitalized for multisystem inflammatory syndrome secondary to COVID‐19 infection 2 weeks prior to the diagnosis of uveitis. At that time, she was treated with corticosteroids, vasopressors, and antibiotics. In contrast to our patient, she had a negative PCR test for COVID‐19 from a nasopharyngeal swab but SARS‐CoV‐2 immunoglobulin G was positive showing a previous COVID‐19 infection. She has successfully treated with topical dexamethasone 0.1% and cycloplegia. This previous case report confirms the concept that uveitis could be a part of multisystem inflammatory syndrome secondary to COVID‐19 infection.

Based on the current knowledge of COVID‐19 infection, various organs could be affected during the acute phase of COVID‐19 infection. The reason is the expression of the ACE 2 receptor by different human tissues, which builds the main route for the virus to enter tissue cells. Different parts of the ocular system might be invaded through this mechanism. Conjunctivitis is the most well‐recognized ocular involvement in COVID‐19 infection.^8^ Alcalde et al.^9^ investigated 17 children with COVID‐19, in which conjunctivitis was the most frequently detected ocular finding (three patients). Two patients had episcleritis, one had retinitis, and one had optic neuritis. Mazzotta et al.^10^ reported acute anterior uveitis and acute bilateral follicular conjunctivitis in a 30‐year‐old female patient with a positive PCR test for COVID‐19. Iriqat et al.^11^ reported three cases of uveitis in 19‐, 29‐, and 62‐year‐old men. The first one had bilateral anterior uveitis, the second one had bilateral intermediate and posterior uveitis, and the last one had right eye iridocyclitis. Rheumatologic tests returned negative for all of them. They were successfully treated with topical and systemic steroids.

We conclude that acute anterior uveitis and optic disk edema could be a manifestation of the acute phase of COVID‐19 infection or occurs as a part of MIS secondary to COVID‐19. Since uveitis could be an eye‐threatening condition, physicians must pay attention to this less‐recognized manifestation of COVID‐19 infection to avoid irreversible complications.

## ETHICAL APPROVAL

Approval was not needed by the local Clinical Research Ethics Committee for case reports.

## AUTHOR CONTRIBUTIONS

**Aria Mohamadzadeh:** Data curation; writing – original draft. **Dena Mohamadzadeh:** Conceptualization; writing – original draft.

## FUNDING INFORMATION

We received no funding.

## CONFLICT OF INTEREST STATEMENT

The authors declare that they have no competing interests.

## CONSENT

Written informed consent was obtained from the patient for publication of this case report and any accompanying images. A copy of the written consent is available for review of the Editor‐in‐Chief of this journal.

## Data Availability

Data are available if requested.
